# Possibilities for examining the neural control of gait in humans with fNIRS

**DOI:** 10.3389/fphys.2014.00204

**Published:** 2014-05-28

**Authors:** Stéphane Perrey

**Affiliations:** Movement to Health (M2H), Montpellier-1 University, EuroMovMontpellier, France

**Keywords:** locomotion control, NIRS, humans, cortical activation, analysis

Although the existence of a central pattern generator system modulated by sensory information has become broadly accepted in the control of gait, many findings indicate that the cortex also plays a role of primary importance in human walking (Miyai et al., 2001; Gwin et al., 2011; Petersen et al., 2012). Examining the neural control of gait in humans requires recording cortical activity during gait. Direct evidence for cortical involvement in human locomotion comes from neuroimaging studies using position emission tomography (la Fougère et al., 2010), electroencephalography (EEG, Gwin et al., 2011) and functional near-infrared spectroscopy (fNIRS, Miyai et al., 2001) methods. Among possible neuroimaging methods, fNIRS is ideally suited to perform brain imaging during walking as it represents several advantages over other methods (Perrey, 2008).

In this opinion article, we concentrate on the possibilities of examining the neural control of gait in humans with fNIRS method. Until now this versatile neuroimaging technique has been used rarely while the subject is moving in neuroscientific research and clinical setting (e.g., Holtzer et al., 2011). While clinical gait evaluation belongs to the neurological examination, very little research on gait with fNIRS measurement from a neurological perspective has been done. Because fNIRS is still at its infancy, it suffers from the lack of standards for a well understanding of signals obtained and signal-processing method. Based on a quick review of recent studies using fNIRS modality for studying gait in humans, this paper aims to address the sensitivity and pitfalls of fNIRS to activation over multiple cortical areas involved in gait control in humans.

Traditional neuroimaging has focused on detecting brain activity in response to a task. However, due to the lack of flexibility of most imaging techniques (e.g., functional magnetic resonance imaging or fMRI), little is known about brain activity during everyday motor tasks and when a patient undergoes gait training. Like fMRI, fNIRS is a non-invasive imaging technique for measuring local variations of hemoglobin concentration changes related to neuronal activity by the phenomenon of neurovascular coupling. By measuring absorption properties of hemoglobin at two or more wavelengths, fNIRS exploits the changes of the wavelength-dependent extinction spectra of the oxygenated (O_2_Hb) and deoxygenated (HHb) form. Although some pitfalls are to consider using fNIRS during gait (i.e., low spatial resolution, inter-subject variability of the hemodynamic response, positioning of the optodes and systemic interference), the advantages of fNIRS, such as non-invasiveness, highly portable make it a promising method for studying the cortical activation patterns associated with whole-body tasks by wearable multichannel fNIRS system (Piper et al., 2014).

In healthy people, the first study using multichannel fNIRS demonstrated significantly increased levels of O_2_Hb in bilateral supplementary motor area (SMA) and primary motor (M1) and somatosensory (S1) cortical regions during treadmill walking (with arm swing) at 1 km/h (Miyai et al., 2001). Walking at 3 and 4 km/h induced evoked hemodynamic responses from the bilateral primary sensorimotor areas (SM1, Suzuki et al., 2004). Running at 9 km/h led to additional oxygenation changes in premotor cortex (PMC) and especially in prefrontal cortex (PFC). While clear SMA and PFC activation changes are also well documented (Holtzer et al., 2011; Koenraadt et al., 2014), changes in speed had little effect on M1 or S1 activity (Suzuki et al., 2004). fNIRS studies have shown that SMA is playing a role in the period prior to the start of gait (Mihara et al., 2007) and for the more difficult task (such as backward walking at 1.6 km/h in Kurz et al., 2012). Although coordinated movement during walking appear relatively effortless, motor commands are important because of the added need for control of stability (Yang and Gorassini, 2006), especially during backward walking (Kurz et al., 2012). Preliminary data from Mazerie et al. (2012) showed that varied terrains (downhill and uphill) activated differently the cortical motor networks (including SM1, SMA, and PFC) than steady-speed treadmill walking due to larger contribution from sensory afferents in walking control and a higher degree of movement difficulty. Besides investigating cortical patterns related to different walking speed and terrain, a verbal cue while walking leads profoundly to different PFC and PMC activation patterns than walking without a verbal cue (Suzuki et al., 2008). This indicates that anticipated adaptations of gait to changes of treadmill speed readily affect regional activations in PFC, SMA, PMC, and SMC. Altogether, an involvement of M1 remains controversial during normal gait in humans (Miyai et al., 2001; Kurz et al., 2012), while the PMC, SMA, and PFC are predominantly involved in adapting to increasing speed and generally during complex gait. These findings indicated that areas involved in planning and allocating attentional resources play a crucial role in controlling locomotion. Hence, fNIRS studies on gait under challenging conditions of walking are likely better suitable to discriminate the involvement of multiple cortical regions. Recently, Koenraadt et al. (2014) did not find a difference in SMA, M1, and S1 activity between precision stepping (challenging condition) and normal walking at 3 km/h but precision stepping placed larger demand on the PFC. Possible discrepancies between the aforementioned studies could originate from different analysis methods, location of the optodes and experimental design. Overall these results highlight that the cortical processing in gait control is influenced by gait parameter (speed, stride-time variability) and cognitive load during the walking task.

Predicting recovery of walking within the context of rehabilitation following stroke is still difficult. We can consider desirable to allow for an adaptation of the optimal rehabilitation strategies not only by behavioral performance but also depending on the patterns of brain activation. The rationale to measure brain activation is that plasticity of the neuronal network entertaining sensorimotor function can be considered the basis of effective rehabilitation. With regard to stroke patients, SMC and PMC activation has been observed during walking (Miyai et al., 2002). Consequently, a rehabilitation training program may be targeted to facilitate motor recovery with early exposure to somatosensory stimulations of these brain regions after stroke. Further the PMC was suggested to be involved in mediating the proximal leg movements and the control of speed of walking in stroke patients (Miyai et al., 2002). A long-term follow-up is still needed to determine how different forms of gait training with improved clinical outcomes influence cortical activation patterns with fNIRS.

Despite feasibility of NIRS for recording brain activation during gait, a number of limitations of fNIRS should be considered. fNIRS is unsuitable for activation of deeper structure than the cerebral gray matter. Further, NIRS suffers from limited spatial resolution (beyond 3–5 mm based on modeling and simulation procedure, Strangman et al., 2002; close to 2–3 cm in practice because NIRS detects near-infrared light scattered and reflected in the brain) and does not enable exact localization of the measured activity within the cortex even if fNIRS time series are closely related to fMRI signals (Muthalib et al., 2013). Correction for measurement error in both optode position and skull reference points (based on standard brain templates) have been recently proposed to overcome this issue (Fekete et al., 2011). Then, the hemodynamic change measured from the scalp may contamine the signal. NIRS provides data both on O_2_Hb and HHb. Surprisingly, there seems to be limited additional information in the two hemoglobin signals. Primary focus is usually on the O_2_Hb measurement due to the better signal-to-noise ratio (relative to HHb) following functional activation (Miyai et al., 2001; Leff et al., 2011); hence a restricted area of statistical significant changes in [HHb] occurs (Sato et al., 2007). Note that brain activation among various brain areas may underlie different patterns of O_2_Hb and HHb changes (Koenraadt et al., 2012) and explain inter-individual variability of fNIRS signals during sensorimotor cortex activation (Sato et al., 2005). Recently Kurz et al. (2012) suggested that HHb should be disregarded for evaluating cortical activation during gait. However, one important problem for the monitoring of brain activation is that extracortical changes (due to systemic changes e.g., in blood pressure or heart rate) are more likely to influence O_2_Hb than HHb (Kirilina et al., 2012). Hence, physiological artifacts induced during gait condition need to be carefully controlled for, especially due to blood flow and hemoglobin changes in the extracortical (i.e., superficial) tissue. Heart rate fluctuations cause changes in the arterial compartment. Because O_2_Hb is representative of the arterial compartment, it is more affected by these systemic fluctuations than HHb which comes mostly from the venous compartment. Kirilina et al. (2012) suggested that looking at the changes [HHb] may allow identification of false positive in NIRS activation maps (i.e., erroneously attributing NIRS responses to cortical changes). Using methods to separate cortical and extracortical signals in NIRS signals include the use of additional short source-detector separation optodes as regressors (Gagnon et al., 2012) and the analysis of the photon time-of-flight distribution in time-domain NIRS (Aletti et al., 2012).

Of note that large body movements during gait may lead to optical fibers displacements on the head, which would translate into a large hemoglobin artifact in the fNIRS signal. Tight fixation of the fibers and the fNIRS probes is crucial while walking. A combination of a customized head cap that holds the fiber holders together with a proper fiber bundle suspension to provide further stability without interfering with the subject's movement is warranted. Different motion artifact techniques (e.g., adaptive filtering Zhang et al., 2009, Kalman filter and independent component analysis) and the use of co-located channels have been proposed for their ability to minimize the effects of physiological motion artifacts in near-infrared imaging (see Robertson and Douglas, 2010).

No standardized methods for fNIRS data analysis have been established yet. Up to date, the only invariant is that different experimental designs require different analysis techniques driven by the underlying neurophysiological mechanism and with a good comprehension of neurovascular coupling. For gait, increased cortical processing is related to large gait parameter changes (Gwin et al., 2011; Kurz et al., 2012) during the stimulation period (e.g., varied terrain and speed) as compared to steady-speed walking conditions. The relevant temporal window for fNIRS signals analysis in detecting brain activity should be determined accordingly. Averaging and baseline correction are conventional signal-processing methods used for the NIRS signal (Derosière et al., 2014) and appears suitable for a block-design for detecting differences in stimuli. During the early phase of locomotor performance, effect size should be calculated to overcome the influence of differential pathlength factors among subjects and brain regions on O_2_Hb and HHb (Suzuki et al., 2004). In the context of gait, the intensity (e.g., speed) and/or complexity of the gait modulates likely the hemodynamic response (Leff et al., 2011). Therefore, temporal window selection where cortical activation is expected to occur should incorporate variable duration for estimating the “true” time to peak of O_2_Hb and the time of nadir of HHb by considering the onset of the stimulus (Leff et al., 2011). Completing this analysis by an evaluation of the activation dynamics (e.g., by an analysis of the slope of the O_2_Hb from onset of the stimulus to peak) might be relevant (Mandrick et al., 2013). Alternatively, individual channel time series of fNIRS data can be reconstructed by using a reference waveform (i.e., trapezoidal function) corresponding to the expected hemodynamic response function (Kurz et al., 2012). Finally, occurrence probability of typical activation pattern has to be evaluated individually, due various sources of noise (see above).

The potential of fNIRS application in the study of human brain activation during gait is promising, motivating further application-specific development toward neuroscience and clinical questions. The investigation of cortical activity by fNIRS presents real advantages especially when measurement in ecologically valid conditions is required. However, neuroimaging of gait is not straightforward and remains difficult. A signal-processing method to extract walking-related components has still to be proposed for fNIRS signals during gait. Also while fNIRS use during gait training in clinical settings might be viewed as an interesting diagnostic tool, many potential confounding variables resulting from disease itself and from extracortical changes need to be carefully controlled.

## Conflict of interest statement

The author declares that the research was conducted in the absence of any commercial or financial relationships that could be construed as a potential conflict of interest.
